# Development and validation of risk prediction model for sarcopenia in patients with colorectal cancer

**DOI:** 10.3389/fonc.2023.1172096

**Published:** 2023-07-27

**Authors:** Ying Zhang, Yongjian Zhu

**Affiliations:** ^1^ College of Nursing, Qingdao University, Qingdao, China; ^2^ Nursing Department, Yantai Yuhuangding Hospital, Yantai, China

**Keywords:** colorectal cancer, sarcopenia, malnutrition, influence factors, nomogram

## Abstract

**Objectives:**

Sarcopenia is associated with a poor prognosis in patients with colorectal cancer. However, the clinical factors that lead to colorectal cancer patients with sarcopenia are still unclear. The objective of this study is to develop and validate a nomogram for predicting the occurrence of sarcopenia and to provide healthcare professionals with a reliable tool for early identification of high-risk patients with colorectal cancer associated sarcopenia.

**Methods:**

A total of 359 patients diagnosed with colorectal cancer from July 2021 to May 2022 were included. All patients were randomly divided into a training (n = 287) cohort and a validation cohort (n = 72) at the ratio of 80/20. Univariate and multivariate logistic analysis were performed to evaluate the factors associated with sarcopenia. The diagnostic nomogram of sarcopenia in patients with colorectal cancer was constructed in the training cohort and validated in the validation cohort. Various evaluation metrics were employed to assess the performance of the developed nomogram, including the ROC curve, calibration curve, and Hosmer-Lemeshow test.

**Results:**

Smoking history, drinking history, diabetes, TNM stage, nutritional status, and physical activity were included in the nomogram for the prediction of sarcopenia. The diagnostic nomograms demonstrated excellent discrimination, with AUC values of 0.971 and 0.922 in the training and validation cohorts, respectively. Moreover, the calibration performance of the nomogram is also excellent, as evidenced by the Hosmer-Lemeshow test result of 0.886.

**Conclusions:**

The nomogram consisting of preoperative factors was able to successfully predict the occurrence of sarcopenia in colorectal cancer patients, aiding in the early identification of high-risk patients and facilitating timely implementation of appropriate intervention measures.

## Introduction

Colorectal cancer is one of the most common malignant tumors in the gastrointestinal tract. Colorectal cancer exhibits a notable global burden, with men ranking it as the third most prevalent cancer and women as the second most prevalent (1). Surgical resection stands as the primary approach for the management of colorectal cancer, epitomizing the cornerstone of therapeutic interventions (2). Owing to the localized impact of postoperative obstruction and malabsorption on intestinal functionality, patients afflicted with colorectal cancer frequently encounter varying degrees of malnutrition, rendering them susceptible to muscular atrophy (3).

Sarcopenia is defined as the age-related loss of skeletal muscle mass plus loss of muscle strength and/or reduced physical performance (4). Sarcopenia exhibits a remarkable prevalence in patients with colorectal cancer. Several studies have revealed a wide-ranging prevalence of sarcopenia in colorectal cancer patients, ranging from 12% to 71%. Regrettably, this critical condition is frequently overlooked, leading to treatment delays and potentially compromising patient outcomes. Sarcopenia exhibits a close association with heightened chemotherapy toxicity, heightened susceptibility to severe complications, and diminished survival rates, underscoring its significance as a prognostic factor in colorectal cancer patients (5, 6). Recent research has unveiled the efficacy of early nutritional and exercise interventions in effectively impeding the progression of sarcopenia, with exercise emerging as a predominant intervention strategy for this debilitating condition (7, 8). Diminished muscle mass, as indicated by the skeletal muscle mass index (SMI), stands as a pivotal diagnostic criterion for identifying sarcopenia, underlining its significance in clinical assessment (4). Computed Tomography (CT) scan is a reference method for evaluating skeletal muscle quality because of its accuracy and reliability (9). Nevertheless, CT imaging, despite its exceptional accuracy, is accompanied by drawbacks such as high costs, the need for skilled operators, and significant exposure to ionizing radiation, posing challenges to its widespread utilization (10). Consequently, the evaluation of sarcopenia in routine clinical practice remains arduous, thereby impeding the formulation of effective strategies for timely identification and subsequent management of this condition.

A concise summary of the influencing factors of sarcopenia in colorectal cancer patients has been provided in certain countries. However, it is important to note that the influencing factors may vary across different cultural backgrounds, leading to heterogeneity in the observed associations (9, 11). In China, researchers have primarily directed their attention towards investigating the influencing factors of sarcopenia in the elderly population, with limited knowledge available regarding its specific determinants among patients diagnosed with cancer. A comprehensive understanding of these factors could aid healthcare providers in prognosticating the overall outcomes of patients, thereby facilitating more informed clinical decision-making. Furthermore, interventions can be implemented to enhance patient outcomes in both the short and long term by targeting the identified risk factors, leading to improved overall prognosis.

Hence, the aim of this study is to develop and validate a nomogram that can effectively predict the occurrence of sarcopenia in patients with colorectal cancer. The proposed nomogram will serve as a valuable tool for healthcare providers, enabling them to implement timely interventions and optimize patient outcomes.

## Methods

### Patients

This study specifically targets patients with colorectal cancer who underwent radical surgery at a tertiary hospital in Yantai between July 2021 and May 2022. The inclusion criteria encompassed the following aspects: ①patients aged 18 years or older; ②patients with a confirmed diagnosis of colorectal cancer based on histology and scheduled for elective surgery; ③patients possessing adequate reading, writing, and language communication skills; ④patients who willingly participated in the study and provided signed informed consent forms, in collaboration with voluntary collaborators. The exclusion criteria comprised the following: ①patients with a prior history of other malignancies; ②non-collaborative individuals (such as those with intellectual disabilities) or those who declined participation in the research; ③patients with physical deformities that would impede muscle strength or physical fitness assessments. Prior to their inclusion in the study, all patients provided informed consent, duly informed that their clinical information would be utilized anonymously for research purposes. This study received approval from the Ethics Committee of Qingdao University (QDU-HEC-2021171), ensuring adherence to ethical guidelines.

### Data collection

To mitigate potential bias, patient data were meticulously collected by referring to pertinent medical records and administering structured questionnaires. All data were securely stored in an electronic database, ensuring the preservation and confidentiality of the information. The following data were collected for each patient: sex, age, BMI, tumor-node-metastasis (TNM) stage, smoking history, drinking history, hypertension, diabetes, stroke, coronary heart disease, total protein, albumin, uric acid, urea nitrogen, calcium, creatinine, creatine kinase, hemoglobin, neutrophil/lymphocyte ratio, Patient Generated Subjective Global Assessment (PG-SGA) and International Physical Activity Questionnaire Short Form (IPAQ-SF). Blood routine and biochemical indexes were taken from fasting venous blood within 24 hours after admission and sent to the biochemical laboratory detection. In accordance with the World Health Organization (WHO) definition established in 1997, a patient was classified as a smoker if they had a history of continuous or cumulative smoking for six months or longer throughout their lifetime. A patient classified as consuming alcohol was defined as someone whose self-reported alcohol consumption was more than once per week.

### Nutrition assessment

In this study, the PG-SGA scale was utilized to comprehensively evaluate the nutritional status of patients diagnosed with colorectal cancer. The PG-SGA scale was divided into two distinct components: patient self-assessment and assessment conducted by medical staff. The first part included weight changes, eating, symptoms, physical function; the second part included the relationship between disease and nutritional needs, metabolic stress needs, physical examination. The first component involved patients self-assessing their nutritional status, denoted as the A score. The second component entailed medical staff evaluating the B score (disease and age), C score (stress), and D score (physical examination). The total score of PG-SGA = A+B+C+D, the higher the score, the worse the nutritional status. According to the score, each patient was divided into three levels: good nutrition (grade A), mild or suspected malnutrition (grade B) and severe malnutrition (grade C).

### Measurement of muscle mass

The measurement of patients’ muscle mass was conducted using the InBody720 body composition analyzer, a device manufactured by Basbeth Company of Korea. This sophisticated equipment allowed for precise assessment of muscle mass in the study participants. The ratio of limb skeletal muscle mass to height square (SMI) (kg/m^2^) showed that the LSMI threshold was 5.7kg/m^2^ for females and 7.0kg/m^2^ for males.

### Measurement of muscle strength and physical fitness

The muscle strength was evaluated by the (Xiang Shan CAMRYEH101) grip strength meter. Subjects were instructed to utilize their dominant hand to perform a minimum of two isometric contraction tests, exerting maximum force during each test. The highest recorded value from these tests was then selected as the maximum reading volume for further analysis. AWGS recommends that male grip strength < 28.0kg and female grip strength < 18.0kg are the diagnostic cutoff values of low muscle strength.

The 6m walking test was used to evaluate the physical fitness of the patients. The time taken by the subjects to walk a distance of 6 meters at their normal speed, without any acceleration or deceleration, was measured on at least two occasions. The average speed during these trials was recorded for subsequent analysis. The walking test was conducted in an indoor corridor, where a measurement scale was prominently marked on the floor surface. All the subjects were tested in the same marked corridor. AWGS suggested that walking speed < 1.0m/s is the diagnostic cutoff value of physical decline.

### Diagnosis of sarcopenia

The diagnosis of sarcopenia in this study followed the updated consensus guidelines established by the AWGS in 2019 (4). The criteria encompassed three fundamental aspects: muscle mass, muscle strength, and physical fitness. Sarcopenia is defined as the concurrent occurrence of reduced muscle mass, diminished muscle strength, and/or impaired physical fitness. For the results of the study, individuals who do not meet these criteria are considered normal.

### Model construction and validation

To establish the training and validation cohorts, random resampling was performed using an 80/20 split. In the training cohort, the prediction model was developed utilizing Logistic regression analysis. The risk factors included in the model were carefully selected based on univariate analysis, which allowed for the identification of potential variables associated with sarcopenia. Subsequently, these significant risk factors were further incorporated into a multivariate logistic regression analysis. Subsequently, a nomogram was constructed based on these selected predictors, allowing for accurate prediction of sarcopenia in patients. The receiver operating characteristic (ROC) curve was constructed to evaluate the discriminative ability of the nomogram model, and the area under the curve (AUC) was calculated as a quantitative measure of its performance. A higher AUC value indicates a stronger discriminatory power of the nomogram in predicting the occurrence of sarcopenia. The accuracy of the nomogram model was evaluated through the construction of a calibration curve and the application of the Hosmer-Lemeshow test. A closer alignment between the prediction calibration curve and the standard curve (Hosmer-Lemeshow test: P > 0.05) indicates a higher level of accuracy and reliability in the model’s predictions.

### Statistical analysis

Statistical analysis was conducted using SPSS Statistics version 26.0 software. The normality of continuous data was assessed using the Kolmogorov-Smirnov test. Continuous data with a normal distribution were presented as mean ± standard deviation (SD) and compared using the Student’s t-test. On the other hand, continuous data with a non-normal distribution were described as median with interquartile range (IQR) and compared using the nonparametric Wilcoxon rank-sum test. Categorical data were presented as frequencies and percentages, and comparisons were made using the Pearson chi-square test or Fisher’s exact test.

## Results

### Demographic and clinical characteristics

From July 2021 to May 2022, a total of 359 patients were enrolled in this study. These patients were randomly allocated to two groups using an 80:20 ratio: the training cohort consisting of 287 patients and the validation cohort consisting of 72 patients. **Table 1** presents the comparison of clinical and pathological characteristics between the training and validation cohorts. The results demonstrate that the two cohorts exhibit similar distributions, thus validating their suitability for use as training and validation cohorts. In the training cohort, the incidence of sarcopenia was determined to be 19.2%, and a comparable incidence was observed in the validation cohort.

**Table 1:** Differences of demographic and clinical characteristics between the training and validation cohorts (n=359).

Variables	Training cohort (n=287)	Validation cohort (n=72)	P
Sarcopenia			0.957
Yes	55 (19.2%)	14 (19.4%)	
No	232 (80.8%)	58 (80.6%)	
Diagnosis			0.077
Colon	138 (48.1%)	43 (59.7%)	
Rectum	149 (51.9%)	29 (40.3%)	
Sex			0.853
Male	184 (64.1%)	47 (65.3%)	
Female	103 (35.9%)	25 (34.7%)	
Smoking history			0.070
Yes	59 (20.6%)	22 (30.6%)	
No	228 (79.4%)	50 (69.4%)	
Drinking history			0.723
Yes	53 (18.5%)	12 (16.7%)	
No	234 (81.5%)	60 (83.3%)	
Diabetes			0.488
Yes	71 (24.7%)	15 (20.8%)	
No	216 (75.3%)	57 (79.2%)	
High blood pressure			0.293
Yes	90 (31.4%)	18 (25%)	
No	197 (68.6%)	54 (75%)	
Coronary heart disease			0.821
Yes	18 (6.3%)	4 (5.6%)	
No	269 (93.7%)	68 (94.4%)	
Stroke			0.286
Yes	17 (5.9%)	2 (2.8%)	
No	270 (94.1%)	70 (97.2%)	
TNM stage			0.161
I	11 (3.8%)	1 (1.4%)	
II	149 (51.9%)	41 (56.9%)	
III	95 (33.1%)	17 (23.6%)	
IV	32 (11.2%)	13 (18.1%)	
Nutritional status			0.107
A	69 (24.0%)	11 (15.3%)	
B	177 (61.7%)	54 (75.0%)	
C	41 (14.3%)	7 (9.7%)	
Physical activity			0.613
Low	56 (19.5%)	13 (18.1%)	
Middle	166 (57.8%)	46 (63.8%)	
High	65 (22.7%)	13 (18.1%)	
Age (years)	63.90 ± 11.22	62.21 ± 11.17	0.252
Total protein (g/L)	62.82 ± 6.53	64.01 ± 6.67	0.169
Albumin (g/L)	36.75 ± 4.38	37.75 ± 4.34	0.084
Calcium (mmol/L)	2.24 ± 0.13	2.26 ± 0.11	0.317
Urea nitrogen (mmol/L)	4.81 (3.85,6.05)	5.03 (3.79,6.59)	0.317
Creatinine (umol/L)	61 (53,69)	59.5 (52,66)	0.377
Uric acid (umol/L)	281.59 ± 101.51	302.88 ± 103.88	0.114
Creatine kinase (U/L)	65 (45,94)	60 (45,91.75)	0.528
Hemoglobin (g/L)	122.97 ± 23.68	126.68 ± 21.67	0.228
BMI (kg/m^2^)	24.16 ± 3.41	24.09 ± 3.23	0.881
Neutrophils/lymphocytes	2.21 (1.54,3.83)	2.19 (1.49,3.84)	0.753

Data are mean±SD, n (%), or median (25–75th percentile) unless otherwise indicate.

### Establishment of prediction model

**Figure 1:**
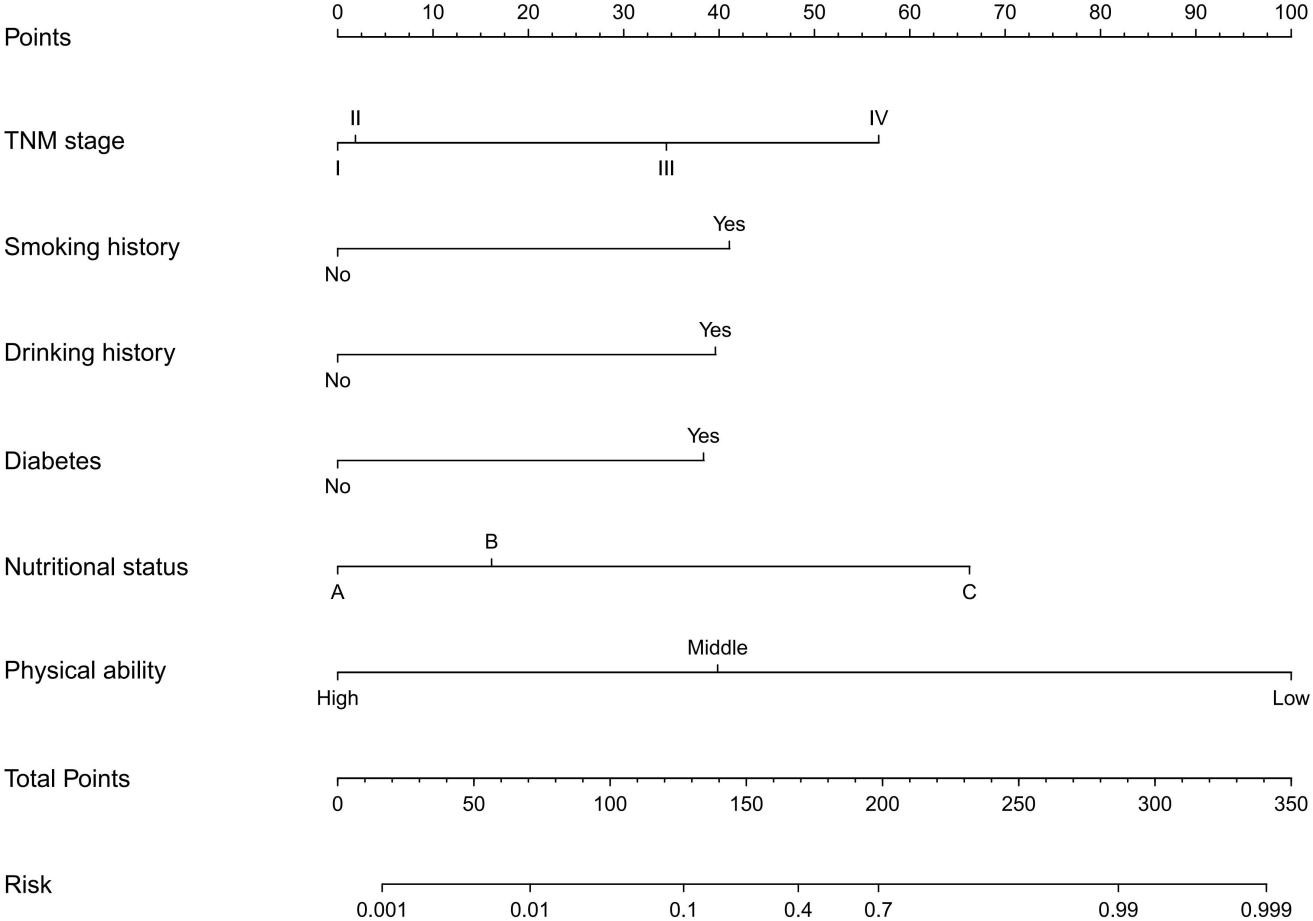
Nomogram to predicting the risk of sarcopenia in colorectal cancer patients.

287 patients in the training cohort were divided into two groups: sarcopenia group (n=55) and non-sarcopenia group (n=232). The results of the univariate analysis revealed that smoking history (P<0.001), drinking history (P<0.001), diabetes (P<0.001), coronary heart disease (P<0.001), TNM stage (P<0.001), nutritional status (P<0.001), physical activity (P<0.001), age (P<0.001), total protein (P<0.001), albumin (P<0.001), calcium (P<0.001), uric acid (P=0.013), creatine kinase (P=0.005), hemoglobin (P<0.001), BMI (P < 0.001) and neutrophils/lymphocytes (P<0.001) were statistically significant between the two groups (**Table 2**). Subsequently, the six variables were included in a multivariate logistic regression analysis. The results of the regression analysis revealed that smoking history, drinking history, diabetes, TNM stage, nutritional status, and physical activity emerged as independent predictors of sarcopenia in patients with colorectal cancer (**Table 3**). Based on the six independent predictors, we developed a nomogram to predict the occurrence of sarcopenia in patients with colorectal cancer (**Figure 1**).

**Table 2:** Univariate analysis of risk factors for sarcopenia in patients with colorectal cancer (n=287).

Variables	Non-sarcopenia (n=232)	Sarcopenia (n=55)	P
Sex			0.392
Male	146 (62.9%)	38 (69.1%)	
Female	86 (37.1%)	17 (30.9%)	
Smoking history			<0.001
Yes	28 (12.1%)	31 (56.4%)	
No	204 (87.9%)	24 (43.6%)	
Drinking history			<0.001
Yes	30 (12.9%)	23 (41.8%)	
No	202 (87.1%)	32 (58.2%)	
Diabetes			<0.001
Yes	40 (17.2%)	31 (56.4%)	
No	192 (82.8%)	24 (43.6%)	
High blood pressure			0.124
Yes	68 (29.3%)	22 (40.0%)	
No	164 (70.7%)	33 (60.0%)	
Coronary heart disease			<0.001
Yes	7 (3.0%)	11 (20.0%)	
No	225 (97.0%)	44 (80.0%)	
Stroke			0.870
Yes	14 (6.0%)	3 (5.5%)	
No	218 (94.0%)	52 (94.5%)	
TNM stage			<0.001
I	10 (4.3%)	1 (1.8%)	
II	141 (60.8%)	8 (14.6%)	
III	61 (26.3%)	34 (61.8%)	
IV	20 (8.6%)	12 (21.8%)	
Nutritional status			<0.001
A	66 (28.4%)	3 (5.5%)	
B	158 (68.1%)	19 (34.5%)	
C	8 (3.5%)	33 (60.0%)	
Physical activity			<0.001
Low	17 (7.3%)	39 (70.9%)	
Middle	151 (65.1%)	15 (27.3%)	
High	64 (27.6%)	1 (1.8%)	
Age (years)	62.37 ± 11.01	70.36 ± 9.73	<0.001
Total protein (g/L)	63.75 ± 6.12	58.88 ± 6.77	<0.001
Albumin (g/L)	37.45 ± 3.95	33.80 ± 4.90	<0.001
Calcium (mmol/L)	2.25 ± 0.12	2.18 ± 0.14	<0.001
Urea nitrogen (mmol/L)	4.69 (3.84,6.01)	5.21 (3.89,6.66)	0.190
Creatinine (umol/L)	61 (53,69)	60.5 (50,69)	0.589
Uric acid (umol/L)	290.01 ± 95.22	246.07 ± 119.14	0.013
Creatine kinase (U/L)	67 (50,96)	49 (34,82.5)	0.005
Hemoglobin (g/L)	125.38 ± 22.86	112.80 ± 24.58	<0.001
BMI (kg/m^2^)	24.87 ± 3.15	21.15 ± 2.80	<0.001
Neutrophils/lymphocytes	2.17 (1.47,3.37)	3.48 (1.70,6.27)	0.001

Data are mean±SD, n (%), or median (25–75th percentile) unless otherwise indicated.

**Table 3:** Multivariate logistic analysis of risk factors for sarcopenia in patients with colorectal cancer.

Variables	β	SE	Wald x^2^	P	OR	95%CI
Smoking history	1.748	0.683	6.556	0.010	5.745	1.507-21.901
Drinking history	1.686	0.745	5.119	0.024	5.398	1.253-23.254
Diabetes	1.633	0.591	7.647	0.006	5.119	1.609-16.288
TNM stage(I)			8.996	0.029		
(II)	0.079	1.699	0.002	0.963	1.083	0.039-30.216
(III)	1.467	1.663	0.779	0.378	4.337	0.167-112.896
(IV)	2.415	1.784	1.832	0.176	11.189	0.339-369.372
Nutritional status (A)			12.829	0.002		
(B)	0.687	0.864	0.633	0.426	1.988	0.366-10.800
(C)	2.820	0.984	8.208	0.004	16.780	2.437-115.519
Physical activity (Low)			20.392	<0.001		
(Middle)	-2.560	0.628	16.644	<0.001	0.077	0.023-0.264
(High)	-4.256	1.250	11.598	0.001	0.014	0.001-0.164
Constant	-3.346	1.921	3.034	0.082	0.035	

### The performance of nomogram in training cohort and validation cohort

**Figure 2:**
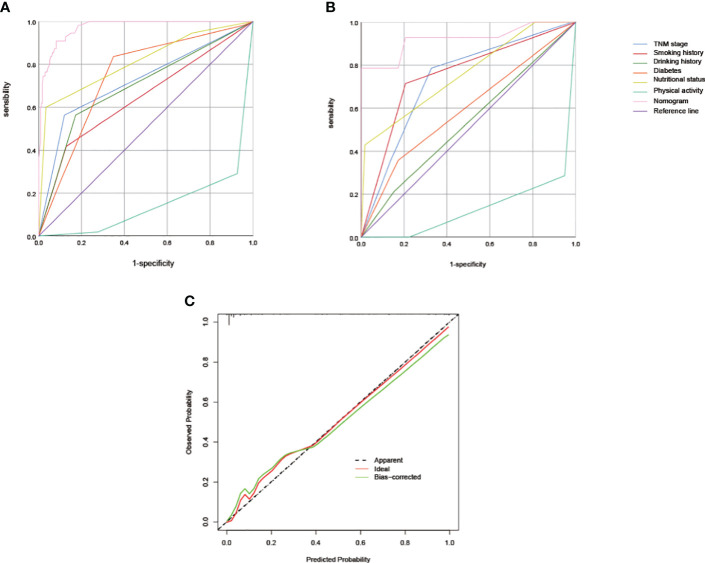
**(A)** The ROC curves of predictive factors and nomograms for predicting sarcopenia in the training cohort (n = 287) **(B)** The ROC curves of predictive factors and nomograms for predicting sarcopenia in the validation cohort (n = 72). **(C)** The calibration curve for risk of sarcopenia in patients with colorectal cancer. ROC, receiver operating characteristic.

To assess the discriminative capacity of the nomogram, we generated ROC curves for both the individual predictors and the nomogram (**Figures 2A, B**). Additionally, we calculated the AUC to quantitatively evaluate the accuracy of each model (**Table 4**). The AUC value in the training cohort was 0.971, and the AUC value in the validation cohort was 0.922. The correction performance of the model was assessed using the Hosmer-Lemeshow test and calibration curve. The Hosmer-Lemeshow test yielded a non-significant result (P = 0.886), indicating that there was no significant difference between the observed probabilities and the predicted probabilities generated by the nomogram. The calibration curve also shows the consistency between the actual probability and the predicted probability (**Figure 2C**).

**Table 4:** Area under the curve for preoperative predictive factors in the training cohort and validation cohort.

Factors	training cohort (95%CI)	validation cohort (95%CI)
Smoking history	0.721 (0.638-0.805)	0.754 (0.603-0.905)
Diabetes	0.696 (0.612-0.779)	0.592 (0.417-0.768)
Drinking history	0.644 (0.556-0.733)	0.530 (0.356-0.703)
TNM stage	0.743 (0.673-0.813)	0.735 (0.592-0.879)
Nutritional status	0.813 (0.741-0.885)	0.760 (0.611-0.909)
Physical activity	0.150 (0.090-0.211)	0.137 (0.019-0.254)
Nomogram	0.971 (0.954-0.988)	0.922 (0.820-1.000)

In addition, in the total population, the sensitivity, specificity, positive predictive value and negative predictive value of sarcopenia prediction were 76.81%, 96.21%, 82.81% and 94.58%, respectively (**Table 5**). Patients in the training and validation cohort had similar results.

**Table 5:** Accuracy of the nomograms to predict sarcopenia.

Factors	training cohort	validation cohort	Total population
**Sensitivity, %**	90.91 (79.29-96.60)	78.57 (48.82-94.29)	88.41 (77.89-94.51)
**Specificity, %**	91.81 (87.31-94.87)	93.10 (82.45-97.77)	92.07 (88.18-94.80)
**Positive predictive value, %**	72.46 (60.19-82.21)	73.33 (44.83-91.09)	72.62 (61.62-81.52)
**Negative predictive value, %**	97.71 (94.43-99.15)	94.74 (84.45-98.63)	97.10 (94.13-98.64)
**Positive likelihood ratio**	11.10 (7.16-17.22)	11.39 (4.26-30.49)	11.15 (7.46-16.65)
**Negative likelihood ratio**	0.10 (0.04-0.23)	0.23 (0.08-0.63)	0.13 (0.07-0.24)

Values are given as percentages (95% CI) or ratio (95% CI).

## Discussion

To the best of our understanding, this study is the first of its kind to examine the ability of preoperative factors to predict the development of sarcopenia in individuals with colorectal cancer. This novel research fills a critical gap in the existing literature and provides valuable insights into the identification and assessment of sarcopenia in this specific patient population. We established a predictive model to predict the condition of sarcopenia. In our study, smoking history, drinking history, diabetes, TNM stage, nutritional status, and physical activity emerged as independent risk factors for sarcopenia. Building upon these significant predictors, we further developed a user-friendly and visually appealing nomogram as a predictive tool. The nomogram exhibited impressive predictive performance, with AUC values of 0.971 (95% CI: 0.954-0.988) in the training cohort and 0.922 (95% CI: 0.820-1.000) in the validation cohort. Furthermore, the calibration curve of the nomogram demonstrated excellent calibration accuracy. Our findings highlight the significant role of readily accessible preoperative factors in accurately predicting sarcopenia. This predictive ability holds great potential for facilitating early interventions and targeted treatments for patients affected by sarcopenia. By identifying individuals at risk, healthcare professionals can initiate timely interventions and implement tailored strategies to mitigate the impact of sarcopenia.

In recent years, there has been an increasing clinical interest in sarcopenia, leading to a growing number of surveys on the prevalence of sarcopenia in colorectal cancer patients. However, several studies have indicated significant variations in the prevalence of sarcopenia among colorectal cancer patients (12–14), which may be attributed to differences in the source of cases. Sun et al. conducted a meta-analysis and found that the prevalence of sarcopenia among the included 5,337 colorectal cancer patients was 46.3% (15). This study used the latest diagnostic criteria for sarcopenia established by AWGS in 2019 and investigated 359 patients with colorectal cancer. The prevalence of sarcopenia was 19.2%, which was lower than the results of the systematic review but higher than that reported by Souza(15%) (9).

Smoking history and drinking history were independent risk factors for sarcopenia in patients with colorectal cancer. In a longitudinal study conducted by Locquet (16), the association between smoking and sarcopenia was examined. The findings revealed that smokers had a significantly higher risk of developing sarcopenia, with a 2.68-fold increased risk compared to non-smokers. These results were further validated through sensitivity analysis, which reinforced the robustness and consistency of the findings on a global scale. Tobacco contains various volatile and soluble components, such as reactive oxygen species (ROS), aldehydes, and reactive nitrogen species (RNS) (17). Upon smoking, these components are introduced into the bloodstream and can subsequently reach skeletal muscle tissues. The presence of these harmful substances in the skeletal muscle microenvironment can contribute to oxidative stress, inflammation, and cellular damage. This may further lead to the development and progression of sarcopenia (17). Analogous to the detrimental effects of smoking, excessive alcohol consumption has been linked to impaired protein metabolism in skeletal muscle. Elevated alcohol intake has been implicated in various detrimental effects on the body, such as urinary incontinence and hepatocyte damage. Additionally, excessive alcohol consumption can stimulate the body to generate high levels of ammonia, resulting in a condition known as hyperammonemia (18, 19). The accumulation of ammonia interferes with the normal processes of protein synthesis and degradation within muscle cells, leading to an imbalance in skeletal muscle protein turnover. This disturbance in protein metabolism may contribute to the development and progression of sarcopenia (20). Furthermore, when individuals engage in both excessive drinking and smoking, the combined impact of these habits on systemic inflammation can exert an additive inhibitory effect on the incidence of muscle protein synthesis (MPS) (19).

The association between diabetes and sarcopenia has been well-established (21). A study utilizing Dual-energy X-ray Absorptiometry (DXA) measurements of skeletal muscle index revealed that individuals with type 2 diabetes had a higher prevalence of sarcopenia compared to the general population. Specifically, 15.7% of patients with type 2 diabetes were found to have sarcopenia, whereas the prevalence in the general population was reported to be 6.9% (22). In our study, 43.66% of diabetic patients developed sarcopenia. In patients with diabetes, the presence of insulin resistance disrupts the delicate equilibrium between muscle protein synthesis and degradation, ultimately leading to a decline in muscle mass. Insulin resistance hampers the uptake and utilization of glucose by skeletal muscles, contributing to a decrease in insulin sensitivity (23). This creates a detrimental cycle (23).

This study showed that there was a correlation between TNM stage and sarcopenia. In our study, we observed patients with stage III/IV disease were found to have a 5.1-fold higher likelihood of developing sarcopenia compared to patients with stage I/II disease. Patients with stage III/IV colorectal cancer exhibit a higher susceptibility to negative nitrogen balance and negative energy balance compared to patients with early-stage disease. This can be attributed to the spread of cancer cells to the surrounding lymph nodes and distant tissues (24). In order to meet the energy needs of the body, the oxidation of non-essential amino acids in skeletal muscle increases, which accelerates the degradation of protein in skeletal muscle and eventually leads to sarcopenia (25). However, the study conducted by Souza showed that there was no difference in the TNM stage (9).

Numerous studies have examined the association between nutritional status and sarcopenia, particularly in patients with colorectal cancer (26). The presence of colorectal cancer increases the susceptibility to malnutrition, which can arise from various factors, including diminished dietary intake, compromised digestive function, and impaired nutrient absorption (27, 28). A study conducted in elderly hospitalized patients during acute post-care revealed a coexistence rate of 15% for malnutrition and sarcopenia (29), which aligns with our own findings. Similarly, a recent systematic review reported a prevalence of 23% for the simultaneous presence of malnutrition and sarcopenia among hospitalized elderly patients (30). Beaudart et al (31) followed up the malnourished elderly in the community for four years and found that the risk of developing sarcopenia/severe sarcopenia (EWGSOP2) tripled during the follow-up period. The association between malnutrition and severe sarcopenia can be elucidated by the insufficient intake of vital nutrients, including vitamin D, protein, and calcium. These nutritional deficiencies have a profound impact on the preservation of muscle mass, subsequently influencing muscle strength and overall physical fitness (32). Nevertheless, it is imperative to establish a causal relationship between malnutrition and sarcopenia, which necessitates longitudinal data on nutritional status and dietary intake for comprehensive investigation.

Physical activity is considered to be the main factor in stimulating muscle protein synthesis, has a protective effect on sarcopenia, and can reduce the probability of developing sarcopenia (33), which is similar to the results of this study. In our study, we observed a substantial association between exercise ability and the development of sarcopenia. Specifically, a remarkable 24.3% of patients with moderate and low exercise ability exhibited sarcopenia, whereas a mere 1.6% of patients with high exercise ability developed sarcopenia. A great deal of evidence has proved that effective exercise intervention can prevent the occurrence and development of sarcopenia (34–36). Exercise interventions encompass both aerobic and resistance exercises, each contributing to varying degrees in augmenting muscle mass in the human body. Especially high-intensity resistance exercise can effectively improve or prevent the decrease of muscle fibers and increase muscle strength (37).Some studies have found that the combination of nutrition and exercise intervention plays an important role in the prevention and treatment of sarcopenia, which can improve the muscle strength of patients more than exercise intervention or nutrition intervention alone (38–40).

Therefore, patients with history of smoking, drinking, diabetes, high tumor stage, malnutrition and low exercise ability should be given corresponding nutrition and exercise intervention according to the actual situation of the patients (41–43).

There are still some limitations in this study. First of all, the current study was conducted at a single center with a relatively small sample size, which may limit the generalizability of the findings. Therefore, it is recommended to expand the sample size in subsequent stages of the study to enhance statistical power and obtain more robust results. Second, it is worth noting that the average BMI observed in our study was relatively low, which may reflect the characteristics of the Asian population. Therefore, additional research is warranted to validate the performance of our nomogram in individuals with higher body mass index, particularly in obese individuals.

In conclusion, we have successfully developed a novel predictive model to assess the risk of sarcopenia in patients with colorectal cancer. Our model highlights the importance of considering factors such as smoking history, drinking history, diabetes, TNM stage, nutritional status, and physical ability as potential risk factors for sarcopenia in this patient population. It is crucial for healthcare professionals to proactively identify individuals at risk for sarcopenia and implement targeted intervention strategies to enhance the quality of life for patients with colorectal cancer. By incorporating this predictive model into clinical practice, we can optimize patient care and improve patient outcomes in this specific population.

## Conclusion

To sum up, a highly accurate prediction model was developed and validated to predict the risk of sarcopenia in patients with colorectal cancer. The model provided a clinical basis for the choice of individualized treatment by identifying the risk of individual sarcopenia. It also provided a new way to improve the quality of life of patients with colorectal cancer.

## Data availability statement

The original contributions presented in the study are included in the article/Supplementary Material. Further inquiries can be directed to the corresponding author.

## Ethics statement

The studies involving human participants were reviewed and approved by Ethics Committee of Medical Science Center of Qingdao University. The patients/participants provided their written informed consent to participate in this study. Written informed consent was obtained from the individual(s) for the publication of any potentially identifiable images or data included in this article. The study was conducted in accordance with the guidelines of the Declaration of Helsinki and approved by the Ethics Committee of the Medical Science Center of Qingdao University (agreement code QDU-HEC-2021171 and 2021-03). All patients have signed informed consent, understanding that the material has educational value, and have agreed to show the material to appropriate professionals and use it in publications.

## Author contributions

YZ conducted data investigation, data analysis and the writing of major articles. YJZ reviewed and revised the first draft. All authors reviewed the manuscript. All authors contributed to the article and approved the submitted version.
